# Development of Monoclonal Antibodies and Antigen-Capture ELISA for Human Parechovirus Type 3

**DOI:** 10.3390/microorganisms8091437

**Published:** 2020-09-19

**Authors:** Keiko Goto, Yutaro Yamaoka, Hajera Khatun, Kei Miyakawa, Mayuko Nishi, Noriko Nagata, Toshikazu Yanaoka, Hirokazu Kimura, Akihide Ryo

**Affiliations:** 1 Department of Microbiology, Yokohama City University School of Medicine, Kanagawa 236-0004, Japan; t176023b@yokohama-cu.ac.jp (K.G.); yutaro.yamaoka@gmail.com (Y.Y.); hkhatun1@gmail.com (H.K.); keim@yokohama-cu.ac.jp (K.M.); mnishi@yokohama-cu.ac.jp (M.N.); 2 Ibaraki Prefectural Institute of Public Health, Ibaraki 310-0852, Japan; eiken1@pref.ibaraki.lg.jp (N.N.); eiken4@pref.ibaraki.lg.jp (T.Y.); 3 Life Science Laboratory, Technology and Development Division, Kanto Chemical Co., Inc., Kanagawa 259-1146, Japan; 4 Department of Health Science, Gunma Paz University Graduate School, Gunma 370-0006, Japan; h-kimura@paz.ac.jp

**Keywords:** human parechovirus, monoclonal antibodies, ELISA, VP0

## Abstract

Human parechovirus type 3 (HPeV3) is an etiologic agent of respiratory diseases, meningitis, and sepsis-like illness in both infants and adults. Monoclonal antibodies (mAbs) can be a promising diagnostic tool for antigenic diseases such as virus infection, as they offer a high specificity toward a specific viral antigen. However, to date, there is no specific mAb available for the diagnosis of HPeV3 infection. In this study, we developed and characterized mAbs specific for HPeV3 capsid protein VP0. We used cell-free, wheat germ-synthesized viral VP0 protein for immunizing BALB/c mice to generate hybridomas. From the resultant hybridoma clones, we selected nine clones producing mAbs reactive to the HPeV3-VP0 antigen, based on enzyme-linked immunosorbent assay (ELISA). Epitope mapping showed that these mAbs recognized three distinct domains in HPeV3 VP0. Six mAbs recognized HPeV3 specifically and the other three mAbs showed cross-reactivity with other HPeVs. Using the HPeV3-specific mAbs, we then developed an ELISA for viral antigen detection that could be reliably used for laboratory diagnosis of HPeV3. This ELISA system exhibited no cross-reactivity with other related viruses. Our newly developed mAbs would, thus, provide a useful set of tools for future research and ensure HPeV3-specific diagnosis.

## 1. Introduction

Human parechoviruses (HPeVs) belong to the *Parechovirus* genus of the *Picornaviridae* family [1,2]. The two serotypes (HPeV1 and HPeV2) of parechoviruses were initially isolated in 1956 from children with diarrhea, and were assigned to the *Enterovirus* genus. However, it became evident that they were genetically distinct from the enteroviruses, and reclassified as the *Parechovirus* genus in 1999. At present, 19 HPeVs are reported and categorized, based on the nucleic acid sequences of the VP1 gene, not on the classification as enteroviruses serotype. HPeV3 was first identified in Japan. HPeV3 was isolated from a stool sample provided by a 1-year-old infant who was experiencing fever, gastritis like symptoms, and transient lower extremity paralysis [3]. HPeV types 1 to 8 are the common identified strains; among them, HPeV type 1, 3, and 6 account for the majority of infectious strains worldwide [4]. Infection with HPeVs is associated with a broad spectrum of clinical manifestations, ranging from respiratory symptoms and mild gastrointestinal illness to sepsis-like diseases, meningitis, and encephalitis in children [5]. While most HPeVs cause mild symptoms in children between 1 and 5 years of age, human parechovirus 3 (HPeV3) is clinically the most important genotype, owing to its association to severe diseases in younger infants under 3 months of age [6,7,8]. HPeV3 infection in infants can trigger a sepsis-like dysregulated host response involving the central nervous system [9,10,11,12]. In cases of acute meningitis or encephalitis, patients might develop abnormal white matter lesions and neurological sequelae, and even death might occur [13,14,15,16,17]. Apnea can occur in children regardless of encephalitis [17]. HPeV3 is known to cause myalgia and myositis in adult patients and a similar pattern is also sporadically seen among pediatric patients. [18,19]. An epidemic of HPeV3 occurs every 3 to 4 years in Japan. As respiratory disease or meningitis cases due to HPeV3 are not subject to notifiable disease surveillance in Japan, the actual number of the patients is not known [1,6,20,21,22,23]. The appropriate diagnosis tool for HPeV3 detection might be able to rule out infectious etiology and avoid unnecessary antibiotics use, which is a given because HPeV3 leads to septic shock-like symptoms. For these reasons, the establishment of a method to detect HPeV3 plays a vital role in healthcare fields. Importantly, HPeV3′s epidemic cycle occurs in summer time and is concurrent with enteroviral infection. Thus, it is essential to develop a detection method that does not cross-react with *Enterovirus*.

HPeV3 contains a small, non-enveloped, single-stranded positive-sense RNA genome of approximately 7.3-kb nucleotides [24,25]. The HPeV3 virion is composed of 60 copies of three structural proteins (VP0, VP1, and VP3) that fit together to form a 28-nm-diameter icosahedral shell around the viral genome [26]. The genome encodes a single polyprotein that, during infection, is subsequently cleaved into all essential capsid components and non-structural proteins [24]. VP0 is an important protein for stabilizing the surface of the viral capsid, and the assembly of HPeV is controlled by multiple interactions of the genome with the capsid, through conserved amino acids in VP1 and VP3 [25].

Although RT–PCR-based diagnostic tests targeting 5′-UTR of the HPeV3 genome were developed for HPeV3 detection in clinical samples, there is currently no diagnostic method for detecting the viral antigens. Recently, Chen et al. generated polyclonal antibodies for HPeV3 VP0, and proposed an immunofluorescence-based diagnostic assay [27]. However, this method requires virus isolation by cell culture and takes several weeks for the identification of viral genotype/serotype. Abed et al. developed a serological enzyme-linked immunosorbent assay (ELISA), using a synthetic peptide from the VP0 protein of HPeVs [28]. Although it can provide a definitive diagnosis, serological test requires paired serum samples from acute and recovery phases, which makes it difficult to diagnose immediately as a point of care testing (POCT). To develop a rapid and effective diagnostic strategy, there is an urgent need to produce highly specific monoclonal antibodies (mAbs) toward HPeV3 antigens.

In this study, we sought to generate mAbs specific to the capsid protein VP0 of HPeV3. We prepared the viral VP0 antigen using the wheat germ cell-free system, which has the advantage of producing properly folded functional proteins [29,30], to immunize mice. As a result, we obtained nine mAb clones for characterization, and thereafter, generated an ELISA system that is specifically able to detect the HPeV3 VP0 antigens.

## 2. Materials and Methods

### 2.1. Construction of Expression Vector

Complementary DNA encoding HPeV3-VP0 (GenBank No. AB084913) was used to generate the expression vector for antigen production with the wheat germ cell-free system. The HPeV3-VP0 open reading frame was amplified by PCR, using the corresponding primer pairs. The amplified fragment was cloned into vector pEU-E01-His-TEV-MCS-N2 (CellFree Sciences, Yokohama, Japan), using restriction enzymes *Xho*I and *Spe*I.

### 2.2. Cell-Free Protein Synthesis

In vitro wheat germ cell-free protein synthesis was carried out as previously described [29,30]. For cell-free protein synthesis, WEPRO7240H wheat extract (CellFree Sciences, Yokohama, Japan) was used in the bilayer translation reaction, as previously described. Synthesized proteins were confirmed by immunoblotting. The His-HPeV3-VP0 (full length, 1–298) protein was synthesized using a Proteomist XE robotic protein synthesizer (CellFree Sciences, Yokohama, Japan) for mouse immunization. The cell-free translation reaction mixture was separated into soluble and insoluble fractions by centrifugation at 18,000× *g* for 15 min. The soluble fraction was mixed with Ni-Sepharose High Performance beads (GE Healthcare, Waukesha, WI, USA) in the presence of 20 mM imidazole. The beads were washed thrice with a washing buffer [20 mM Tris-HCl (pH 7.5), 500 mM NaCl] containing 40 mM imidazole. His-HPeV3-VP0 was then eluted in another washing buffer containing 500 mM imidazole. Amicon Ultra centrifugal filters (Millipore, Bedford, MA, USA) were used to concentrate the purified His-HPeV3-VP0. Protein concentration was determined using the Bradford method, with bovine serum albumin (BSA) as a protein standard.

### 2.3. Monoclonal Antibody Production

Immunization of BALB/c mice and generation of anti-HPeV3-VP0 mAb-producing hybridomas were carried out as previously described [29,30]. Briefly, His-tagged full-length HPeV3-VP0 protein was injected into the footpad of the BALB/c mice, using keyhole limpet hemocyanin as an adjuvant. Four weeks later, the spleen cells were isolated and fused to the myeloma cell line, SP2/O, using polyethylene glycol 1500 (PEG 1500). Monoclonal antibodies in the hybridoma culture supernatant were tested using ELISA with His-tagged recombinant HPeV3-VP0 protein. Isotype determination was performed using Isostrip mouse monoclonal antibody isotyping kit, following the manufacturer’s instructions (Roche Diagnostics, Basel, Switzerland).

### 2.4. Cell and Virus Culture

Vero cells were grown in DMEM containing 10% FBS. HPeV3 was provided by Dr. Masaki Takahashi (Iwate Prefectural Institute of Public Health). HPeV3 was propagated in Vero cells and quantified by qRT–PCR. The sequence information was as follows: 5′-GTAACASWWGCCTCTGGGSCCAAAAG-3′ (Forward primer), 5′-GGCCCCWGRTCAGATCCAYAGT-3′ (Reverse primer), and 5′-VIC- CCTRYGGGTACCTYCWGGGCATCCTTC-BHQ-3′ (Probe).

### 2.5. Immunoblotting

Wheat germ-synthesized recombinant His-tagged HPeV3-VP0 proteins were separated by 10% SDS–PAGE in running buffer (250 mM glycine, 25 mM Tris, 0.1% SDS). The separated proteins were transferred to a PVDF membrane (Millipore). The membranes were washed with blotting buffer TBST (TBS containing 0.05%-Tween 20), and then blocked for 1 h at room temperature in 5% non-fat powdered milk in TBST. Thereafter, the membranes were incubated overnight with generated hybridoma supernatant (1:50 dilution in TBST) at 4 °C. Next, after washing thrice in TBST, the membranes were incubated for 1 h at room temperature with anti-mouse IgG–HRP secondary antibody (1:10000 dilution in TBST). Finally, after washing thrice in TBST, the target protein was detected with the Immobilon Western Chemiluminescence detection system (GE Healthcare) using Fluor Chem FC2 (Alpha Innotech Corp. Tokyo, Japan).

### 2.6. Epitope Mapping

For epitope mapping, we prepared deletion mutants of HPeV3-VP0 using PCR mutagenesis with template vector pEU-His-HPeV3-VP0, followed by wheat germ cell-free protein synthesis. The proteins were analyzed by immunoblotting using our generated mAbs.

### 2.7. Multiple Sequence Alignment

To examine the amino acid variability among the VP0 proteins of other HPeV genotypes, VP0 sequences for HPeV1–8, 14, and 17–19 were accessed from the GenBank and aligned with the HPeV3-VP0 sequence using the MEGA software.

### 2.8. Affinity Measurement of mAbs

K_D_ values were determined by Bio-Layer interferometry (BLI) using Octet RED96 (ForteBio, USA). Anti-mouse IgG Capture biosensor tips (AMC, ForteBio) were loaded with 20 µg/mL of mAb #8 and #39 for 5 min, in PBS containing 0.1% BSA and 0.01% Tween20. The association of recombinant VP0 at concentrations of 50, 25, 12, and 5 nM for #8 mAb, and 14, 7, 3.5, and 1.75 nM for #39 mAb, was measured for 5 min, followed by a 10-min-long dissociation phase. All measurements were corrected for baseline drift by subtracting a reference well. The operating temperature was maintained at 30 °C. Data were analyzed using a 1:1 binding model with global fitting algorithms in the ForteBio data analysis software.

### 2.9. Selection of the Optimal Pair of mAbs for Sandwich ELISA

Each mAb was diluted in 50 mM of carbonate buffer (pH 9.6) to a concentration of 10 μg/mL, and then added to an ELISA plate (AGC TECHNO GLASS, Shizuoka, Japan). To immobilize the antibodies, the plate was incubated overnight at 4 °C. Wells were blocked with PBS containing 2% (w/v) skim milk for 1 h at room temperature (RT). After three washes with PBS containing 0.05% (v/v) Tween-20 (PBS-T), 100 μL of antigen protein (8 ng/mL) diluted with PBS-T or blank (PBS-T alone) was added, and the mixture was incubated for 60 min at RT. After three washes with PBS-T, 100 μL of each mAb, conjugated with horseradish peroxidase (HRP), was added into each well and incubated for 60 min at RT. Antibody labeling was performed using the Peroxidase Labeling Kit—NH2 (Dojindo Laboratories, Kumamoto, Japan). After three washes with PBS-T, 100 μL of ABTS substrate solution (Kirkegaard and Perry Laboratories, Washington, DC, USA) was added and the mixture was incubated for 30 min at RT. Absorbance at 405/490 nm was measured on GloMax Discover System (Promega), and the signal-to-noise ratio (S/N) was calculated.

## 3. Results

### 3.1. Generation of mAbs to Target HPeV3-VP0 Protein

**Figure 1:**
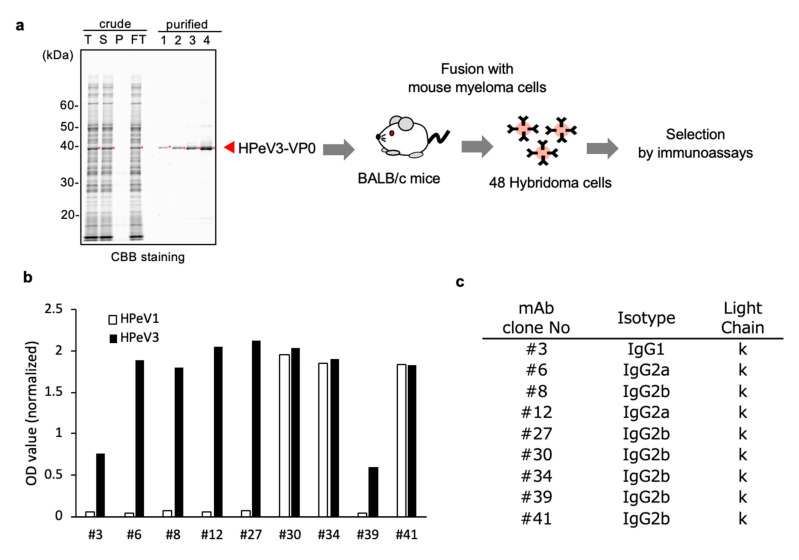
Production of anti-HPeV3 mAbs using wheat germ cell-free synthesized VP0 antigen. (**a**) Schematic representation of hybridoma cell production to generate anti-HPeV3 mAbs using the recombinant Histidine-tagged HPeV3-VP0 (His-VP0) protein produced by the wheat germ cell-free system, and then purified using nickel-chelated Sepharose beads. Each protein fraction was analyzed by SDS–PAGE and visualized by CBB staining. Red dots and arrow indicate the target protein. T—total fraction; S—supernatant; P—precipitate; FT—flow-through; and purified fractions 1–4. Purified His-HPeV3-VP0 antigen was injected into BALB/c mice. After 4 weeks, the spleen cells of immunized mice were fused with myeloma cells and 48 hybridoma clones were established. (**b**) Out of these 48 clones, nine exhibited relatively high reactivity to antigen proteins, as revealed by ELISA. We used HPeV1 and HPeV3 VP0 proteins as antigens. (**c**) Isotyping of selected mAbs-producing hybridoma clones.

For generation of mAbs, we produced N-terminal His-tagged full-length VP0 protein of HPeV3 as an antigen. As a result, HPeV3-VP0 protein was produced with high aqueous solubility (Figure 1a). The protein was subsequently purified using Ni–Sepharose beads, followed by elution with imidazole. BALB/c mice were then immunized with the purified protein. After 4 weeks of immunization, the mice splenocytes were isolated, fused with myeloma cells, and hybridomas were produced. As a result, 48 stable hybridomas were generated and designated as #1 to #48. Among these 48 clones, nine were selected (#3, #6, #8, #12, #27, #30, #34, #39, and #41), based on the reactivity in ELISA to the target antigens VP0 proteins derived from HPeV1 and HPeV3 (Figure 1b). Isotype analysis revealed that #3 mAb belongs to IgG1, kappa isotype, #6 and #12 mAbs belong to IgG2a, kappa isotype, while the others belongs to IgG2b, kappa isotype (Figure 1c).

### 3.2. Epitope Mapping of Anti-HPeV3-VP0 mAb

**Figure 2:**
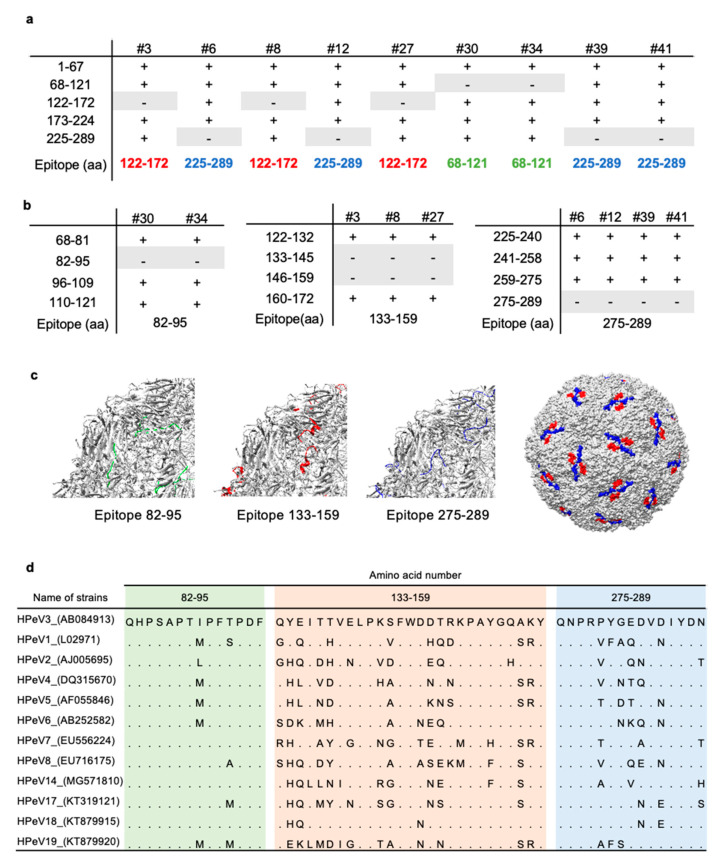
Epitope mapping of mAbs. (**a**,**b**) Deletion mutants of VP0 were produced by wheat germ cell-free protein synthesis. Reactivity of each mAb to the deletion mutants was evaluated by immunoblotting. + and – indicate positive and negative detection, respectively. (**c**) Position of epitopes in a structural model of HPeV3 VP0 (PDB ID. 6GV4). Epitope localizations of the various mAbs are highlighted in different colors. (**d**) Multiple alignment of sequences in HPeV VP0 proteins in the epitope region.

To demonstrate the antibody-binding sites within the antigen, we next performed epitope mapping. For epitope mapping, we produced five deletion mutants of VP0 and performed immunoblotting analysis. We found that our newly developed mAbs recognized three distinct domains in HPeV3 VP0: #30 and #34 mAbs bind to 68–121 amino acid (aa), #3, #8, and #27 mAbs bind to 127–172 aa, and remaining four mAbs (#6, #12, #39, and #41) bind to 225–289 aa within C-terminal region of the VP0 protein of HPeV3 (Figure 2a). We further created deletion mutants for a more precise epitope determination for these mAbs, and found that #30 and #34 mAbs bind to 82–95 aa, #3, #8, and #27 mAbs bind to 133–159 aa, and #6, #12, #39, and #41 mAbs bind to 275–289 aa (Figure 2b). We next examined whether the antigenic epitopes were located on the surface of HPeV3-VP0. The UCSF Chimera software revealed that, except for #30 and #34, the binding regions of all mAbs were located on the molecular surface of VP0 protein (Figure 2c). The binding region of mAbs #30 and #34 was relatively conserved among the analyzed HPeVs (Figure 2d).

### 3.3. Cross-Reactivity of Anti-HPeV3-VP0 mAbs

**Figure 3:**
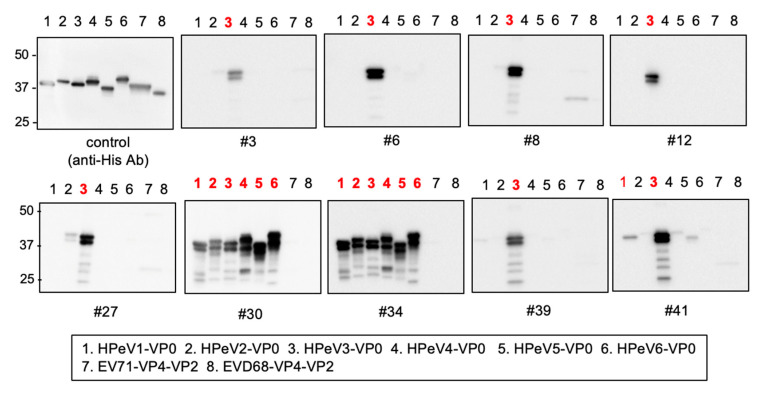
Cross-reactivity of anti- HPeV3-VP0 mAbs. Cross-reactivity of selected mAbs to related HPeVs. His-tagged VP0 proteins of HPeVs were synthesized by a wheat germ cell-free system and the reactivity of mAbs was then assessed by immunoblotting analysis. The numbers associated with lanes showing positive bands are highlighted in red.

We next investigated the specificity of our newly developed mAbs. For this, we created VP0 proteins encoded by the six different HPeV genotypes and VP4-VP2 proteins derived from Enteroviruses 71 and D68. Immunoblotting analysis showed that six mAbs (#3, #6, #8, #12, #27, and #39) react specifically with HPeV3 VP0, while three mAbs (#30, #34, and #41) showed some cross-reactivity to other HPeVs (Figure 3). No mAbs showed cross-reactivity to enterovirus VP4-VP2 proteins.

### 3.4. Development of Antigen-Capture ELISA for HPeV3 VP0

**Figure 4:**
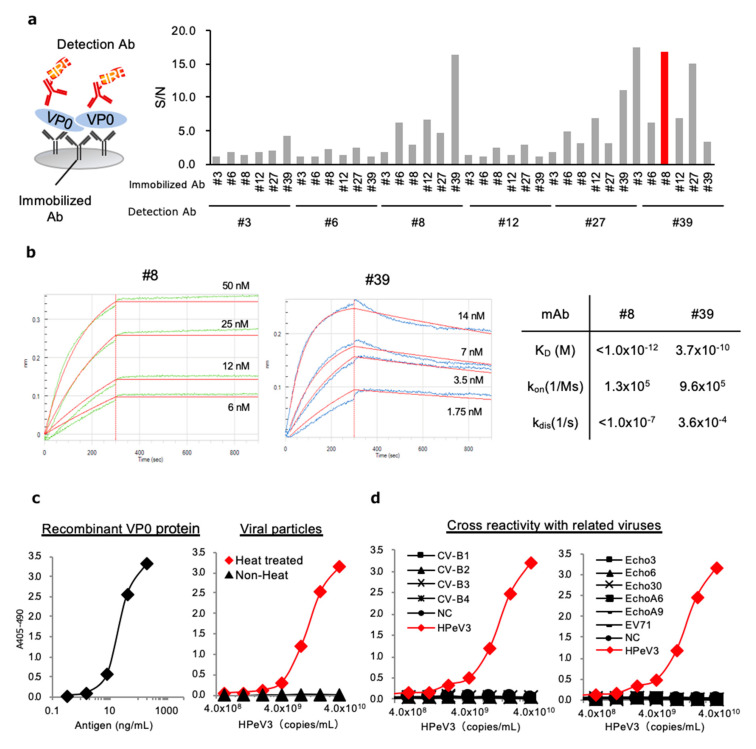
Development of antigen-capture ELISA for HPeV3 VP0. (**a**) Thirty-six mAb pairs were tested for sandwich ELISA. Signal-to-noise (S/N) ratio for antigen detection by each of the 36 combinations was calculated in the presence of 8 ng antigen vs. blank. (**b**) K_D_ value, k_on_, and k_dis_ of #8 and #38 mAbs. (**c**) Detection limit of ELISA for HPeV3 recombinant VP0 proteins (left) and viral particles (right). (**d**) Cross-reactivity with related viruses of the HPeV3 ELISA system.

Sandwich ELISA systems with highly specific matched antibody pairs are commonly used to detect and quantify viral antigens in immunoassay. Hence, we next determined the optimal pair of mAbs for antigen-capture using ELISA, by evaluating all possible combinations of immobilized and labeled mAbs. Among the 36 possible pair combinations, only the #8 and #39 mAb pair represented a combination of antibodies specific for HPeV3-VP0 (Figure 4a). Therefore, we selected this combination for further analysis.

To characterize the equilibrium dissociation constant (K_D_) of the selected antibodies and the target VP0 antigen, we used the BLI Octet Assay System. K_D_ for antibody–antigen binding in mAbs #8 and #39 was calculated as < 1 × 10^−12^ and 3.72 × 10^−10^, respectively, suggesting that both mAbs showed high binding affinity to the HPeV3-VP0 (Figure 4b).

We next performed antigen-capture ELISA with recombinant VP0 protein and virions released into the cell-culture supernatant of the HPeV3-infected cells. Using the optimal antibody pair (#8 and #39 mAbs) identified above, we determined the detection threshold for antigen recognition by antigen-capture ELISA. Our results revealed that our system was highly sensitive to the recombinant antigen, capable of detecting the protein at a concentration of 3 ng/mL (Figure 4c, left). In parallel, we investigated the detection limit of ELISA for the HPeV3 virion. This ELISA system could detect heat-treated HPeV3, but not non-heated virions, and its detection limit of the system was 1 × 10^9^ copies/mL (Figure 4c, right). We also found that this ELISA system exhibited no cross-reactivity with enteroviruses (Figure 4d)

## 4. Discussion

HPeV3 is increasingly being highlighted as a potentially severe viral infection in neonates and young infants. Therefore, there is an urgent need to develop assays for early diagnosis of HPeV3 infection for reducing inappropriate antimicrobial use, unnecessary investigations, and prolonged hospitalization. It is also likely to lead to follow-up for potential complications in infants who are severely affected [1]. In this context, a real-time PCR-based molecular test to detect virus from patients was recently developed [31]. However, PCR tests are extremely sensitive and need extensive controls, whereas antigen detection by mAbs has the advantage of the relative ease of sample handling and the use of less stringent procedures. Specific mAbs could then be used to develop a rapid test such as ELISA. In this study, we sought to generate specific mAbs and develop an ELISA test for the detection of HPeV3 VP0 antigen.

HPeV3 genome encodes for three structural proteins, namely VP0, VP3, and VP1, which assemble to create the virus particle [10]. Among these, VP0 was identified as an antigenic determinant and it might be relatively more useful for diagnostic purposes, due to a higher level of sequence conservation [32]. Furthermore, it possesses high immunogenicity [7]. Therefore, the selection of VP0 as an antigen is both practical and reasonable. In addition, the mAb quality was determined mostly by preparation of high-quality antigen. Here, we used a wheat germ, cell-free protein production system for synthesizing recombinant VP0 proteins, as this system produces properly folded, soluble, and biologically active native proteins similar to those expressed in mammalian cells [29,30,33].

In our current study, we newly produced nine different mAbs that recognized HPeV3-VP0 as an antigen. We then performed epitope mapping of our generated mAbs, as identification of the epitope is a key step in the characterization of monoclonal antibodies [34]. Based on the epitope analysis, the mAbs were able to recognize three different areas of HPeV3-VP0 and specify 12–14 aa length epitopes within the HPeV3-VP0. Interestingly, two mAb clones (#30 and #34) exhibiting cross-reactivity to VP0 proteins of other HPeVs, bound a distinct epitope (82–95 aa), which partially overlapped with the recognition site for the polyclonal antibody (79–99 aa) created by Chen et al. [27]. Nevertheless, we obtained mAbs specific to HPeV3, which recognized sites 133–159 aa and the 275–280 aa site of VP0, which was not reported earlier. Moreover, we also developed an ELISA for detecting HPeV3 antigen using two of our newly generated HPeV3-specific mAbs (#8 and #39), as mAb-based ELISA is highly specific and sensitive towards viral antigen detection [35]. Our Octet assay suggested that both mAbs show a high binding affinity to the full-length HPeV3-VP0 recombinant protein. VP0 protein can be folded into the correct native structure and is likely to form the capsid-like structure via oligomerization. In this situation, #8 mAb could bind various sites on polymerized VP0 proteins, as a result of an allosteric effect or “avidity”, owing to which the K_D_ value of #8 mAb was calculated to be much lower than that of #39 mAb.

Our newly developed ELISA system requires heat treatment of HPeV3 to detect VP0 antigen. Based on previous studies [7], the binding area of mAb #39 was estimated to localize to the interface between VP0 and VP3. On the other hand, three-dimensional modeling of viral particles revealed that the antigen-recognizing sites of mAbs #8 and #39 in VP0 protein are proximally located. Moreover, a previous study showed that Glu285 (in the epitope region of #39) and Ser28 (in the VP0 protein of HPeV3) bind together by a hydrogen bond [24]. Thus, a possible explanation for our observation is that heat treatment helps antigen retrieval for the interface between VP0 and VP3, resulting in efficient access of mAbs to the epitopes.

According to a previous study [36], an increase in antibody-binding capacity was exhibited when glycosylation of capsid protein was removed. Based on this finding, we carried out pretreatment by resecting the connection between O-linked and N-linked glycosylation of HPeV3. However, there was no obvious effect on antibody recognition in our sandwich ELISA assay (data not shown), indicating that glycosylation might not affect the antigenicity for mAb recognition.

The detection limits of our newly developed sandwich ELISA were 3 ng/mL for recombinant VP0 protein and 1 × 10^9^ copies/mL for viral particles, respectively. Assuming that one viral particle contains a single copy of a viral genome, there are 60 copies of VP0 protein per viral particle. For a viral particle detection sensitivity of 1 × 10^9^ copies/mL, the VP0 protein detection sensitivity is presumed to be 3 ng/mL. Therefore, we conclude that the detection sensitivity for the recombinant VP0 protein and the virus particles was almost equivalent, suggesting the feasibility of our method for use in actual clinical settings.

Other than the ELISA method, several antigen-detecting tests using an antigen–antibody interaction are now available. For instance, the multi-array technology using electrochemiluminescence immunoassay (ECLIA) and chemiluminescent enzyme immunoassay (CLEIA) can provide several hundred times more sensitivities than the conventional ELISA method [37]. Another example is an influenza-testing kit using a highly sensitive immunochromatographic detection method based on silver amplification [38]. The limitation of sensitivity might be overcome by utilizing these sophisticated technologies combined with our monoclonal antibodies for HPeV3 VP0 antigen. Furthermore, we can potentially improve the detection sensitivity by altering the second antibody to recognize the poly-HRP complex [39] or by adding other antibodies, which can target VP1 or VP3 proteins.

In summary, we utilized the wheat germ cell-free protein production system to synthesize the HPeV3 VP0 protein and produced mAbs that could specifically detect HPeV3 but not other HPeVs. We further explored the feasibility of these mAbs in terms of their utility in various immunological applications. To the best of our knowledge, the HPeV3 VP0 antibody as well as the ELISA-based viral detection system reported here is the first of its kind ever reported. With the implementation of more sophisticated applications, our newly developed mAbs could be useful for further development of diagnostic methods for HPeV3 infection.
